# The Role of Interventional Radiology for the Treatment of Hepatic Metastases from Neuroendocrine Tumor: An Updated Review

**DOI:** 10.3390/jcm9072302

**Published:** 2020-07-20

**Authors:** Maxime Barat, Anne-Ségolène Cottereau, Alice Kedra, Solène Dermine, Lola-Jade Palmieri, Romain Coriat, Raphael Dautry, Lambros Tselikas, Philippe Soyer, Anthony Dohan

**Affiliations:** 1 Department of Radiology, Cochin Hospital, AP-HP, 27 rue du Faubourg Saint- Jacques, 75014 Paris, France; alice.kedra@gmail.com (A.K.); raphael.dautry@aphp.fr (R.D.); philippe.soyer@aphp.fr (P.S.); anthony.dohan@aphp.fr (A.D.); 2 Université de Paris, 75006 Paris, France; annesegolene.cottereau@aphp.fr (A.-S.C.); solene.dermine@aphp.fr (S.D.); lolajade.palmieri@aphp.fr (L.-J.P.); romain.coriat@aphp.fr (R.C.); 3 Department of Nuclear Medicine, Cochin Hospital, 27 rue du Faubourg Saint- Jacques, 75014 Paris, France; 4 Department of Gastroenterology, Cochin Hospital, 27 rue du Faubourg Saint- Jacques, 75014 Paris, France; 5 Department of Interventional Radiology, Gustave Roussy Cancer Campus, 114 rue Edouard Vaillant, 94805 Villejuif, France; lambros.tselikas@gustaveroussy.fr

**Keywords:** neuroendocrine tumors, neoplasm metastasis, embolization, chemoembolization, brachytherapy

## Abstract

Interventional radiology plays an important role in the management of patients with neuroendocrine tumor liver metastasis (NELM). Transarterial embolization (TAE), transarterial chemoembolization (TACE), and selective internal radiation therapy (SIRT) are intra-arterial therapies available for these patients in order to improve symptoms and overall survival. These treatment options are proposed in patients with NELM not responding to systemic therapies and without extrahepatic progression. Currently, available data suggest that TAE should be preferred to TACE in patients with NELM from extrapancreatic origin because of similar efficacy and better patient tolerance. TACE is more effective in patients with pancreatic NELM and SIRT has shown promising results along with good tolerance. However, large randomized controlled trials are still lacking in this setting. Available literature mainly consists in small sample size and retrospective studies with important technical heterogeneity. The purpose of this review is to provide an updated overview of the currently reported endovascular interventional radiology procedures that are used for the treatment of NELM.

## 1. Introduction

Neuroendocrine tumors (NETs) are still rare tumors although their incidence trends to increase with 6 to 7/100,000 patients per year [1]. They derive from embryonic neural crest tissue and consequently may have a ubiquitous presentation. Most of them originate from the gastroenteropancreatic tract or the lung [2]. However, the primary site remains unknown in 13% of patients [3]. The five-year overall survival (OS) rate widely depends on patient age and sex, primary tumor site, stage of the disease, pathological differentiation, and histological grade [3]. Synchronous distant metastases are present at the time of diagnosis in 20% to 25% of patients, but this prevalence reaches up to 40% during the follow up with the occurrence of metachronous metastases [4]. The presence of distant metastases is independently associated with a poor prognosis [3,4].

In total, 80% of patients with metastatic NETs have liver metastases and the liver is the single metastatic site in 50% of patients (i.e., solitary liver metastases). Solitary liver metastases mostly originate from the gastroenteropancreatic tract [4]. The prognosis of NET liver metastases (NELMs) mostly depends on the extent of liver parenchyma involvement and hepatocellular failure due to liver parenchyma replacement by tumor tissue [5]. In patients with only one or a few NELMs, curative strategies, such as surgery or percutaneous thermal ablation, may be indicated. On the other hand, for patients with multiple, bilateral, or unresectable NELMs, palliative treatments are aimed at reducing tumor volume and endocrine secretion in order to improve symptoms, biological abnormalities, patient quality of life, as well as progression-free survival (PFS) [5].

Treatment of solitary NELM requires a decision during multidisciplinary meetings with liver surgeons, oncologists, radiation oncologists, endocrinologists, radiologists, interventional radiologists, and nuclear medicine physicians. The “toolbox” of available therapeutic options is large and includes pharmacological and non-pharmacological therapies but, because of the rarity of NETs, only a few treatments have been evaluated in large randomized controlled trials (RCTs) [6]. Most of the studies are retrospective with a limited number of patients [6].

The aim of this narrative review was to provide an updated overview of the current intra-arterial treatments used for the treatment NELMs.

## 2. Transarterial Embolization and Transarterial Chemoembolization

### 2.1. Rational of Transarterial Therapies

**Figure 1:**
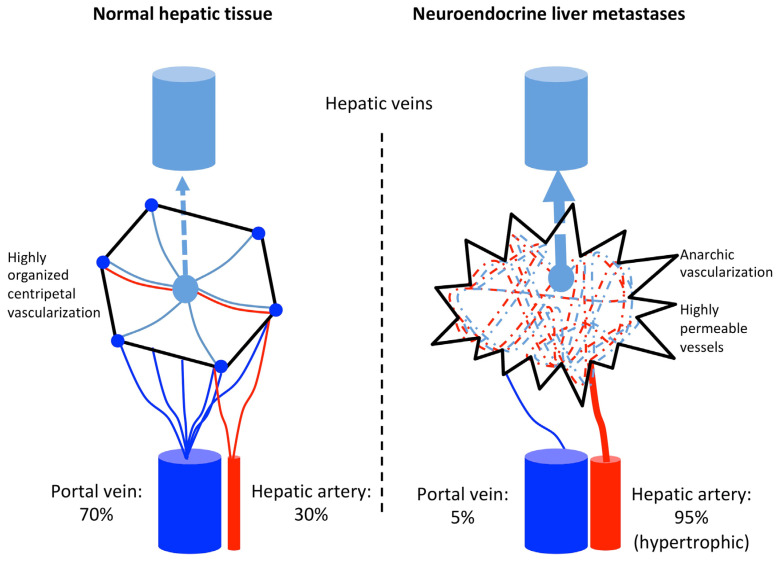
Schematic representation of hepatic vascularization with comparison between normal hepatocytes and liver metastases.

Transarterial embolization (TAE) (i.e., embolization alone without drug infusion or the so-called “bland embolization”) is aimed at occluding small caliber arteries feeding the NELMs and inducing tumor ischemia and necrosis. This treatment is widely performed in other malignant hypervascularized liver tumors, such as hepatocellular carcinoma (HCC) [7]. The basic concept behind this strategy is that NELM vascularization mostly depends on the arterial system whereas normal adjacent liver is mostly supplied by the portal venous system (Figure 1) [8]. Consequently, occlusion of small distal arteries feeding the NELMs is expected to induce tumor necrosis with minimal damage to the non-tumorous adjacent liver parenchyma. Historically, this treatment of NELM was performed surgically with temporary or definitive liver dearterialization in the 1950s, but this approach has been abandoned [9].

NELM progression implies an important blood flow and induces a tumor neoangiogenesis process that is demonstrated during dynamic contrast-enhanced imaging [10,11]. More than 95% of NELMs are hyper enhancing during the arterial phase on contrast-enhanced ultrasonography (CEUS). The remaining 5% of NELMs have a necrotic component and are less sensitive to arterial occlusion [12]. On computed tomography (CT) or magnetic resonance imaging (MRI), imaging findings are quite different, with a high rate of NELMs appearing hypoattenuating or hypointense during the arterial phase, mostly because of a lower temporal resolution compared to CEUS [13].

Transarterial chemoembolization (TACE) associates targeted intra-arterial infusion of chemotherapy followed by arterial embolization. TACE is aimed at increasing the local concentration of cytotoxic drugs inside the tumor and decreasing the systemic concentration and subsequent adverse effects by comparison to standard systemic chemotherapy [14]. Arterial embolization is performed after drug infusion and induces NELM ischemia and reduces drug washout from NELMs compared to infusion alone [14].

During TACE, the drug can be delivered using Lipiodol^®^ (Guerbet) (i.e., conventional TACE (cTACE) during which the chemotherapy is mixed with iodized poppy seed oil) or with drug-eluting beads (DEBs) TACE.

### 2.2. TAE or TACE?

Only a few studies, mostly retrospective, have compared TAE and TACE in NELMs. Some studies found a trend of better improvement of tumor burden and symptoms for TAE compared to TACE but without significant differences and similar patient tolerance between both methods [15,16]. Other studies reported a trend of longer progression-free survival (PFS) with TACE compared to TAE without significant difference but a better tolerance with lower occurrence of adverse events for TAE [17,18]. However, these studies included NELMs from different origins and no sub-group analysis was performed between the different primaries.

Gupta et al. retrospectively compared 69 patients with gastroenteric NETs and 54 patients with pancreatic NETs [19]. Patients with gastroenteric NETs had a greater response rate with TAE than with TACE and patients with pancreatic NETs had a better tumor response rate with TACE. These researchers reported a higher but not significant rate of complications for patients treated with TACE compared with those treated with TAE (20% vs. 12%, respectively; *p* = 0.31) [19]. One prospective randomized study including NELMs from midgut NETs found no differences in terms of PFS and the complication rate between TAE and TACE [20].

The ongoing randomized embolization trial for NELMs (RETNET) is aimed at comparing bland embolization, Lipiodol^®^ chemoembolization, or DEB chemoembolization, with 60 patients per arm. The results of this trial are not yet available [21].

### 2.3. Materials for Embolization

In many studies, the choice and the possible use of absorbable gelatin particles or calibrated non-absorbable particles devices remain at the discretion of the interventional radiologist [22,23,24].

When first described in 1977 by Allison et al., hepatic artery embolization was performed using sterile absorbable gelatin sponge particles (Sterispon^®^, Allen & Hanburys Ltd, London, UK) with dimensions ranging from 1 to 5 mm^3^ [25]. These authors reported persistent arterial occlusion on angiograms performed 6 weeks after TAE but did not evaluate other outcomes [25]. Further studies reported hepatic artery embolization with absorbable gelatin powder (i.e., slurry) with similar results [8,26,27]. However, gelatin powder is no longer used because it is associated with a high risk of complication due to non-targeted embolization [28].

The use of calibrated polyvinyl alcohol (PVA) particles was first described in 1986 [29]. PVA was used to obtain better results because of a more distal embolization; however, these authors did not perform a comparison for tolerance, tumor response, or overall survival (OS) with gelatin sponge [29]. In a retrospective study including 160 patients with NELM treated with TAE, Zener et al. compared outcomes of 83 patients treated with particles <100 μm to those of 77 patients treated with particles >100 μm and found a better initial tumor response rate for patients treated with <100 μm particles but no differences in the OS and complication rate [30].

Definitive embolization using cyanoacrylate mixed with Lipiodol^®^ (Laboratoire Guerbet, Roissy Charles de Gaulle, France) was reported to perform a complete, distal, and permanent embolization [31,32]. However, this approach raised several concerns because no repeat embolization was possible and two early deaths were reported in 29 patients (7%) [31,32]. Of note, one patient died because of extensive hepatic necrosis despite no alterations in portal perfusion [32]. Other embolic agents, such as ethylene vinyl alcohol copolymer (Onyx^®^, EV3, Irvine, CA, USA) are available for embolization, but their use has not been reported for TAE and TACE [33].

### 2.4. How to Perform TACE?

**Figure 2:**
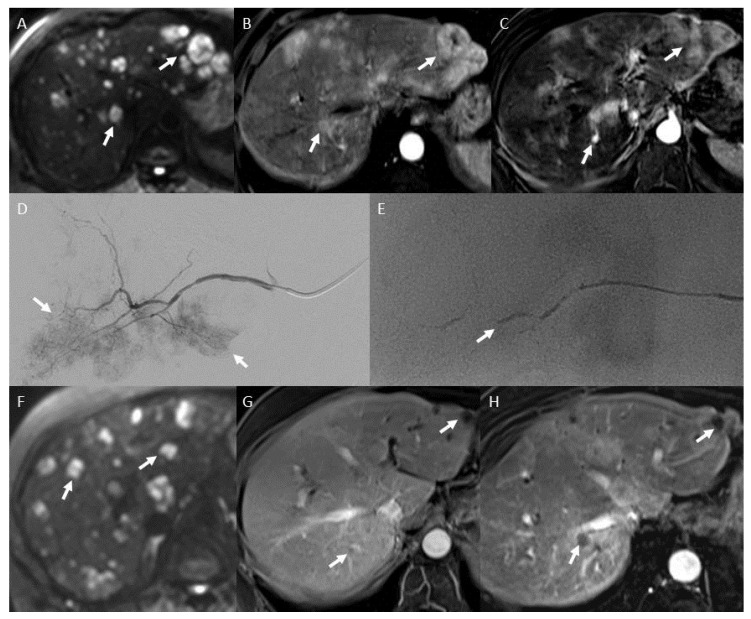
A 71-year-old woman with neuroendocrine tumor from undefined primary and liver metastases with important carcinoid syndrome and liver pain related to diffuse liver involvement. Pre-embolization diffusion-weighted magnetic resonance (MR) image in the axial plane, (**A**), T1-weighted MR image after intravenous administration of a gadolinium chelate during the arterial phase without (**B**) and with subtraction (**C**) show multiple, bilateral liver metastases with restricted diffusion (arrows), and hyperenhancement during the arterial phase (arrows). Patient had 6 sessions of conventional transarterial chemo-embolization under general anesthesia using a mixture of iodized oil (Lipiodol^®^) and doxorubicin and final embolization using calibrated 300–500 µm microspheres. Initial angiogram of right anterior hepatic artery shows multiple arterial tumor blush (arrows) (**D**) and final angiogram shows proximal occlusion of the right anterior hepatic artery (arrow) (**E**). Post-embolization diffusion-weighted MR image in the axial plane (**F**), T1-weighted MR images obtained after injection of gadolinium chelate during the arterial phase without (**G**) and with subtraction (**H**) show multiple, bilateral liver metastases with restricted diffusion (arrows), and hypointensity of most of metastases during the arterial phase (arrows).

To date, no prospective clinical trials have compared different drugs and doses for NELMs. Drugs used for TACE are similar to those used for systemic chemotherapy of primary NET; of these, doxorubicin or streptozocin are the most frequently used [34]. The first drugs used for TACE were emulsified with Lipiodol^®^, resulting in the so-called “conventional” TACE (cTACE) (Figure 2). The first drug used was epirubicin for the treatment of gastroenteric NELM [35]. Since different drugs, associations, and dosages have been tested for cTACE, of these, cisplatin alone or in association with doxorubicin and vinblastine or mitomycin C are the most widely used [36,37]. In a retrospective non-controlled study, including 48 patients with NELMs, Vogl et al. compared mitomycin C to a combination of mytomycin C and gemcitabine for cTACE and found no differences between the two groups in terms of OS and tolerance [38].

DEB TACE uses calibrated microparticles loaded with cytotoxic drugs. These devices were developed with the theorical advantage that the chemotherapeutic agent and the embolization agent are the same, making the procedure easier and shorter [39,40]. Reported results and complication rates were similar to those reported with other therapeutic agent with 80% of partial response and a PFS ranging between 12 and 15 months [39,40]. However, the use of DEB-TACE in patients with non-cirrhotic liver, because of a lower portal blood supply and a higher arterial supply of non-tumoral liver, is associated with an increased risk of biliary injury and liver infarction (odds ratio (OR) = 6.628 (95% CI: 3.699–11.876); *p* < 0.001) and increased risk of liver necrosis compared to cTACE (OR = 35.2 (95% CI: 8.41–147.36)) [41,42]. A phase II study including 13 patients treated with DEB-TACE found a high rate of biliary complications, leading to interruption of the trial [43]. This is particularly true for particles with caliber >300 μm [42]. Makary et al. retrospectively compared outcomes after cTACE and DEB-TACE in patients with NELMs and found a better tolerance for patients treated with cTACE compared to those treated with DEB-TACE [44]. They also reported a better clinical improvement rate for patients treated with cTACE (54%) compared to those treated with DEB-TACE (30%) (*p* < 0.001) but found no differences for the biological or radiological response. As a limitation, however, no comparison of OS was performed [44].

### 2.5. Tolerance and Side-Effects

A large French survey of self-evaluation reported poor or very poor tolerance of TAE and TACE in 50% of treated patients with NELMs [45]. Adverse events are described using the common terminology criteria for adverse events in five grades from mild asymptomatic (grade 1) to death (grade 5) [34,46,47]. In general, perprocedure tolerance of patients is low, with pain occurring during arterial occlusion either with TAE or TACE. To overcome this serious limitation, general anesthesia is usually performed [30,34,48]. Intra-arterial injection of lidocaine is another option to decrease the need for antalgic drugs and decrease hospital stay length [49,50].

The most frequent side-effect of TACE in patients with NELMs is the post-embolization syndrome that associates vomiting, abdominal pain, and fever. It occurs in all patients and is not considered as a complication. The first series described post-embolization syndrome after TACE in all patients with important symptoms that could last up to 2 weeks [8,27,28]. Embolization restricted to one hemi-liver or less (one or two liver segments) improves tolerance with a similar occurrence rate but with a shorter duration of symptoms and lower complication rate [23,37,51]. Nausea and vomiting occur more frequently than fever and pain and can be prevented using per procedure administration of antiemetic drugs [48]. When repeat TACE sessions are performed, the post-embolization syndrome decreases over time [29,52].

Serious adverse events (grade ≥ 3) occur in less than 10% of TACEs in patients with NELMs [37]. A comparative retrospective study found no significant difference in the rate of adverse events between TACE (20%) and TAE (12%) (*p* = 0.31) [19]. Acute carcinoid syndrome may occur in 10% to 32% of patients without clearly identified predictors [37,53,54]. It is the consequence of high blood release of vasoactive substances and may induce hemodynamic instability, malignant hypertension, meningeal hemorrhage, cardiac arrhythmia, and ultimately cardiac failure [23,44,55,56]. Other complications include liver ischemia, non-targeted embolization, and complications due to angiography.

#### 2.5.1. Liver Ischemia

Segmental liver necrosis occurs in 1% to 6% of TAE of TACE in patients with NELMs. Identified risk factors are the use of DEB >300 μm, portal vein thrombosis, and bile duct dilatation [42]. Extensive liver necrosis and death was reported after TAE in one of two patients with the use of cyanoacrylate [32]. Liver abscess occurs in 2% of patients after TACE regardless of the indication [57]. The most important risk factor for liver abscess is bilioenteric anastomosis. In a series of 397 TACEs, Kim et al. reported six liver abscesses in seven patients with bilioenteric anastomosis [57]. When a history of bilioenteric anastomosis is excluded, patients with NELM have a five times increased risk of liver abscess than those treated with TAE for other types of liver tumor [51,57]. Delayed ischemic cholangitis after TAE or TACE has rarely been reported [58].

#### 2.5.2. Non-Targeted Embolization

Non-targeted embolization may occur in 34% of TAE [59]. Ischemic cholecystitis due to cystic artery embolization represents 72% of complications due to non-targeted embolization [15,23,31,36,37]. Gastric or duodenal ulcers, pancreatitis, rectal bleeding, and right adrenal gland necrosis have been reported less frequently [48,60,61,62,63,64]. It is thus recommended to identify the cystic artery origin and perform embolization downstream of its origin [59].

#### 2.5.3. Other Complications

Aspecific complications of arterial approach and iodinated contrast injection remain possible [65,66].

Thrombocytemia, cutaneous rashes, or alopecia have been reported in a few patients treated with TACE for NELM [43,58]. No studies have compared the occurrence rates between drugs. Urinary infections, pulmonary embolism, and alteration of mental status after procedures were reported and seem to be associated with the post-procedural hospital stay length and at a lesser degree with technical parameters [30,47,52]. One patient developing a syndrome of inappropriate secretion of antidiuretic hormone following TAE with hyponatremia and several dermatologic complications has been reported [67,68].

#### 2.5.4. Mortality Related with TAE and TACE in NELMs

Post-procedure mortality after TAE and TACE ranges from 0% to 8% with most deaths related to toxic carcinoid syndrome or liver failure [63,66,69]. This rate is higher for TAE and TACE procedures performed in an emergency setting [47].

### 2.6. Contraindications

For patient safety, platelet blood count >100,000/mL and normal coagulation parameters are required [70]. A leukocyte blood concentration >2000/mm^3^ is recommended [23]. Chronic renal insufficiency with a glomerular filtration rate <30 mL/min is a contraindication to intra-arterial injection of iodinated contrast agent and consequently to TAE and TACE. [37]. Similarly, a high risk of post-embolic acute hepatic failure and carcinoid syndrome should delay the intervention in patients with decompensated liver disease and cardiac failure [24,37].

An excessive liver involvement by NELM is a relative contraindication, with a threshold ranging from 50% to 90% [62,69]. However, this may be balanced by the possibility to perform repeat, hyperselective, and sequential treatments to avoid complications. Similarly, prior bilioenteric anastomosis is a relative contraindication because infectious complications may be prevented by prophylactic antibiotic therapy similar to those used for percutaneous thermal ablation [71]. Then, portal vein thrombosis (acute or chronic) is a contraindication to TAE and TACE in order to avoid hepatic necrosis after arterial occlusion [54].

### 2.7. Therapeutic Response to TAE and TACE in NELMs

No large prospective study has been performed to evaluate the therapeutic response to TAE and TACE. Therapeutic response can be estimated by summarizing the largest retrospective published studies. An improvement of symptoms is observed in 60% to 90% of patients [16,23,54,72]. The mass effect due to liver involvement by NELMs decreases in 100% of patients [37,63].

Radiological response is mostly evaluated using response evaluation criteria in solid tumors (RECIST) algorithm depending on the largest tumor dimension measured in a strict axial plane or modified RECIST (mRECIST) for the most recent studies that only consider the largest diameter in the axial plane of persistent tumor enhancement [73,74]. Considering these different criteria, tumor changes are classified as complete response (CR) when all NELMs disappeared, partial response (PR) when the regression is >30%, stable disease (SD) when the tumor variation range between −30% and +20%, and progressive disease (PD) when the tumor progression is >20% or when new NELMs are identified [73,74]. In most studies, no CR was reported, PR was observed in 50% to 90% of patients, and PD in 0% to 10% of patients [37,54,72]. Approximately 50% of patients with pre-existing biological abnormalities have normalization or decrease of these abnormalities [32,48,62]. The mean duration of these biological improvements ranges between 11 and 20 months, but repeat TAE or TACE may result in a longer duration [23,30,37,63].

Regarding OS, heterogeneity in the design of published studies do not allow firm conclusions to be drawn. The largest and most recent studies report OS ranging from 12 to 84 months after TAE or TACE [55,75,76]. When sub-group analyses are performed to compare pancreatic NELMs and gastroenteric NELMs, TAE is associated with a better OS compared to TACE in patients with gastroenteric NELMs [19]. On the opposite, TACE is associated with a better OS in patients with pancreatic NELMs [19]. Osborne et al. suggested that TAE was less effective for symptoms reduction and OS than surgical cytoreductive therapy [72]. However, this study was retrospective, non-randomized, and patients in the TAE group had a higher rate of poor prognosis factors with unknown primary tumor site, higher liver involvement, and a higher rate of non-resectable primary tumor [72]. Strosberg et al. tried to improve the therapeutic response of TAE using a combination of antiangiogenic drug (sunitinib) in a phase II single arm [55]. However, no subsequent phase III study was published to date. In patients with a tumor burden over 75%, TACE improves symptoms in 85% of patients, biological markers in 80%, and quality of life in most of them and remains a therapeutic option in this specific population despite an OS lower than that in the general population [44,77].

### 2.8. Prognostic Factors

Several prognostic factors of tumor response have been suggested. They include clinical, procedure, biological, and imaging parameters.

#### 2.8.1. Clinical Parameters

A pancreatic primary, an unknown primary site, high tumor grade (i.e., tumor proliferation index marker Ki-67 (Ki67) > 20%), non-resection of the primary NET, liver involvement > 50%, and male gender are associated with a poor OS [19,47,54,60,69,78]. Patients with an Eastern Cooperative Oncology Group (ECOG) score ≥1 have a poorer OS than those with an ECOG score <1 [60,79]. A pre-treatment tumor progression is associated with worst outcomes [55].

Management of patients with NELMs from grade 3 NET (i.e., Ki67 > 20%) is difficult and some authors recommend an aggressive multimodal locoregional management including surgery, thermal ablation, trans-arterial treatments of NELM, and systemic chemotherapy to improve OS and patient quality of life [80].

After TACE, a better tumor response is observed when liver involvement by NELM is between 30% and 50% [81]. When extrahepatic metastases are present, despite a worst prognosis compared to patient with solitary NELM, some authors suggest that TACE may be useful to control symptoms with an improvement in 79% of patients [78,82,83].

Patients with pre-existing malignant carcinoid syndrome have a poorer prognosis after TAE than those without malignant carcinoid syndrome [84]. A body mass index >25 kg/m^2^ is associated with an improved response rate after TACE [85].

#### 2.8.2. Procedure Parameters

NELMs of the caudate lobe (i.e., segment I) have a lower radiological response to transarterial therapy than those in other segmental locations (12% vs. 82%, respectively) with consequent lower OS (46 vs. 87 months; *p* = 0.031) [56]. When several sessions of TAE or TACE are performed, responses after following sessions are similar to the first one for clinical improvement and biological response [26,62]. However, when NELMS favorably respond to the first session, a repeat session improves the OS [26,62].

#### 2.8.3. Biological Prognostic Factors

An elevated pancreastatin serum level (>5000 or 10,000 pg/mL) is associated with a higher risk of periprocedural mortality and poorer long-term OS after TACE [23,79]. A pre-treatment albumin blood level <35 g/L and elevated alkaline phosphatase serum level are associated with poorer OS and PFS after TACE at multivariate analysis [24,86]. The post-operative drop of the 5-hydroxyindolacetic acid level 2 to 4 weeks after TAE is more important in patients with radiological response at 3 months compared to those with stable disease [32].

#### 2.8.4. Pre- and Post-Treatment Imaging Prognostic Factors

Pre-TACE arterial enhancement of NELM on computed tomography (CT) is a predictor of tumor response and prolonged OS [85]. A low Lipiodol^®^ uptake by NELM after TACE is associated with poorer prognosis but is a subjective criterion and was not evaluated for the interobserver reproducibility [66]. Considering quantitative imaging, the difference in enhancing tumor burden between MRI examinations performed in the month before and after TACE using a response cut-off of 50% was associated with a lower OS in non-responders (16.7 months vs. 84.3 months; *p* < 0.01) [79]. Similarly, arterial enhancement >45% and venous enhancement >73% of NELM on MRI in the 6 weeks before TACE are independent predictors of longer OS (OR 2.73; 95% CI 1.45, 5.15; OR 0.32; 95% CI 0.17–0.63; OR 0.35; 95% CI: 0.17–0.72, respectively) and hepatic PFS (HR 2.30, 95% CI: 1.38–3.84; OR 0.46, 95% CI: 0.25–0.84; OR 0.36, 95% CI 0.19–0.57, respectively) [87].

### 2.9. Patients Selection: Role of Interventional Radiologist

The therapeutic decision should be discussed in a dedicated multidisciplinary meeting [88]. Ideal candidates have a non-pancreatic NET with a resected primary tumor site, no extra-hepatic metastases, and a liver involvement between 30% and 50%. However, some benefits may be observed in other patients and all patients should be discussed individually. The interventional radiologist should take care to select the patient, plan the intervention, and exclude potential contraindication. During a dedicated pre-interventional consultation, physical examination should search for history of bilioenteric anastomosis or sphincterotomy, adverse events to iodinated contrast agent, and clinical signs of decompensated liver disease, such as ascites and encephalopathy. Biological blood tests should be performed to evaluate liver function, renal function, and hemostasis. Then, at least a liver-dedicated CT or MRI examination should be performed to evaluate arterial anatomy, extension of liver involvement, portal vein patency, and biliary duct dilatation. Thoracic-abdomen-pelvic CT is indicated to look for extrahepatic metastases from NET.

## 3. Selective Internal Radiation Therapy (SIRT)

### 3.1. Principles and Rationale for SIRT

SIRT consists in a transarterial deposition of a radioactive source inside the tumors [89]. As for TAE and TACE, this is possible because NELMs have a predominant arterial vascularization [8].

Most SIRTs are performed using yttrium 90 (^90^Y), a pure ß-emitter with a physical half-life of 96 h and a mean delivered energy of 0.937 MeV [89]. Beta radiations have a mean penetration in tissues of 2.5 mm and a maximum one of 10 mm; consequently, there is no radiation of the patient environment after the treatment. ^90^Y is obtained by ß transformation of the strontium 90 (mean energy: 0.534 MeV; half live: 26.79 years), a product of the fission of a uranium 235 atom or neutron bombardment of an 89-Y atom in a reactor. They are vectorized using resin (diameter range: 20 µ to 60 µ; activity per particle, 50 Bq) or glass (diameter range: 20 to 30 µm; activity per particle, 2500 Bq) particles. All are larger than the liver capillary diameter.

Another device, loaded with holmium-166 (^166^Ho), has recently been made available [90]. It is a ß-emitter with a half-life of 26.8 h and a mean delivered energy of 1.801 MeV. Radiations have a mean tissue penetration of 4 mm and a maximum of 8.7 mm. Sphere diameter ranges from 15 to 60 µm. Holmium has the advantages of being highly paramagnetic, allowing quantification with MRI and having a small part of γ-ray (0.081 MeV, 5.8%) emitted that may be used for nuclear imaging [91]. It is produced by neutron irradiation of ^165^Ho [92].

When injected selectively, particles will distribute evenly in vessels and stop in distal arteries homogeneously in the tumor without passage in the systemic circulation and consequently no irradiation of healthy organs [89]. It is possible to inject a high dose of radiation directly in the tumor [93]. SIRT is not performed with further arterial embolization.

### 3.2. How to Perform a SIRT

As for other treatments of NELMs, SIRT has to be decided during dedicated multidisciplinary meetings with nuclear physicians. SIRT is mostly performed in the half liver at once with a complementary session 4 to 6 weeks later for bilateral NELMs [94].

**Figure 3:**
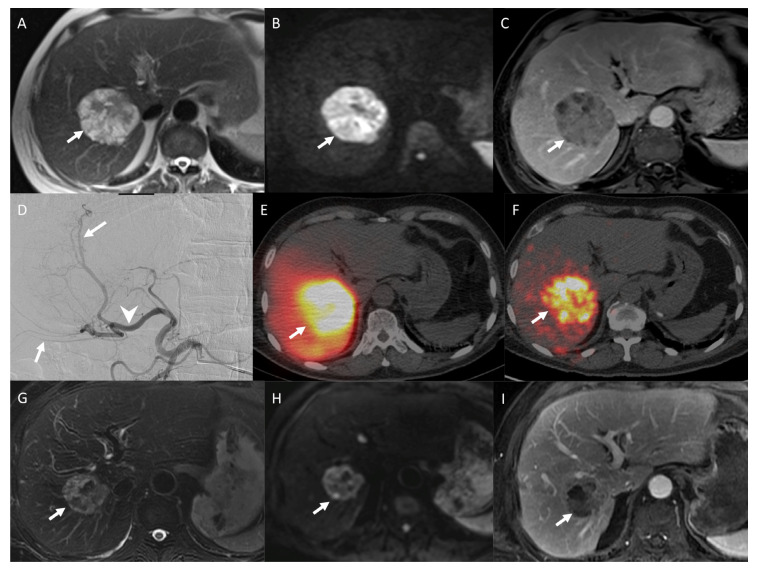
An 80-year-old woman midgut neuroendocrine tumor grade I (Ki67 = 1%), treated 10 years ago with surgery. She had an exacerbation of symptoms resistant to octreotide. MRI examination obtained before selective internal radiation therapy (SIRT) shows a single, hyperintense, and heterogeneous metastasis of the right liver (arrow) on T2-weighted image (**A**), marked diffusion restriction on high b-value diffusion-weighted image (arrow) (**B**), and heterogeneous enhancement on T1-weighted MR image obtained after intravenous administration of a gadolinium chelate during the arterial phase (arrow) (**C**). Arteriogram of common hepatic artery shows two feeding arteries (arrows) originating from the right branch of the hepatic artery (arrowhead). The injection point was defined 15 mm upstream to arterial bifurcation (**D**). Pre-treatment scintigraphy after injection of macroaggregated albumin labeled with 99m-Technetium shows an important uptake by metastasis (arrow), a low uptake by adjacent healthy liver, and no non-target embolization (**E**). Post-SIRT scintigraphy confirmed these results, with uptake by metastasis (arrow) (**F**). One year after SIRT, MRI shows a decrease in lesion size (2.5 versus 6 cm) (arrow), on T2-weighted image (**G**), a decrease in diffusion restriction (arrow) (**H**), and no more hyperenhancement on T1-weighted MR image after injection of a gadolinium chelate during the arterial phase (arrow) (**I**). Patient has no recurrent disease during the first two years after SIRT. Then, she had a complementary treatment by lutetium Lu 177 DOATATE (Lutathera^®^, Novartis, Basel, Switzerland). She is still alive to date.

A work-up is required, consisting in the preparation of the liver to avoid extrahepatic non-target embolization and plan the doses that will be injected. Angiography of the liver is first performed with catheterization of the hepatic artery and tumor feeding arteries. Then, coiling of extrahepatic threatened arteries originating from intrahepatic arterial branches may be performed. Coiling can be performed to occluded falciform artery, cystic artery, arteries from the pancreaticoduodenal arcade, the right gastric artery, or vascular abnormalities, such as arterio-venous fistulas [95]. The systematic coiling of the gastroduodenal artery is no longer recommended because selective catheterism of the hepatic artery and a distal injection of SIRT agent is enough to prevent off-target embolization of the gastroduodenal artery in most procedures [96]. At this step, a dose of 100 to 150 MBq of 99 m technetium-labeled macroaggregated albumin (^99m^Tc-MAA) is injected in a well-defined injection point followed by a single photon emission computed tomography (SPECT) to evaluate the tumor-delivered dose, normal liver-delivered dose, and extrahepatic perfusion, especially the rate of hepatopulmonary shunt (HPS). Work-up should be performed in the 2 weeks before SIRT [97]. If the work-up is non-satisfying, a new one has to be performed before treatment. During the treatment, arterial catheterization is performed and ^90^Y microspheres are administered at the same injection point as that of the therapy work up. Few studies have reported the ratio between the radiation doses prescribed and those actually delivered. When reported, the dose delivered-to-plan rate is over 99% [95,98]. One to 24 h after SIRT, a Bremsstrahlung SPECT imaging is performed to confirm intrahepatic activity, detect extrahepatic activity, anticipate radioembolization-induced extrahepatic side-effects, and calculate the dose delivered to NELMs and healthy hepatic parenchyma (Figure 3) [99].

### 3.3. Complications of SIRT

Specific complications of SIRT mostly include radiation-induced complications. Radiation pneumonitis is a rare but dreadful complication. It may occur when HPS is >10% and induces an interstitial diffuse pneumonitis with restrictive syndrome 1 to 6 months after SIRT [100]. It may be prevented by a reduction of dose from 20% to 40% in patients with HPS >10%. SIRT is contraindicated when HPS is >20% [100].

Liver toxicity is a constant adverse event but mostly asymptomatic [101]. Radioembolization-induced liver disease (REILD) may occur after 0% to 13% of SIRTs [101,102]. It is a potentially life-threatening complication with liver damages characterized by jaundice and ascites, which appear between 4 and 8 weeks after SIRT [103]. Mortality of REILD may reach up to 30% [102]. A radiation dose-to-liver volume ratio >0.8 GBq/L, an elevated dose to normal liver (>50 Gy), pre-existing liver disease, previous chemotherapy, whole liver treatment, extensive tumor burden, and elevated bilirubinemia (>2 g/dL) are identified risk factors of REILD [97,102,103,104,105,106]. The liver complication rate could change between different devices available, but no comparative studies were performed with this goal [94,107]. Morphological signs of cirrhosis and portal hypertension were reported in 16% of patients after SIRT and may occur in 50% after bilateral treatment [94,108]. Post-SIRT liver decompensation is most frequent after bilateral treatment and in patients with pre-SIRT clinical progression [107]. Bile duct dilatation occurs in 20% of patients after bilateral SIRT [108]. Post-SIRT long-term complications are rare and mostly consist in low-grade and intermittent biological complications [94,108]. For hepatocellular carcinomas (HCCs), SIRT seems to be associated with a lower failure rate than TACE [109].

The most frequent radio-induced hematological complication of SIRT is lymphocytopenia, which occurs in 6.7% and thrombocytemia in 3% [110]. Other complications are not specific of SIRT and mostly consist in arterial catheterism-related complications and non-targeted embolization resulting in radiation-induced cholecystitis, ulcer, and gastritis in 1% to 4% of patients [100,111,112]. Risk of liver abscess in patients with previous history of biliary intervention is lower than for TACE/TAE [113]. In a systematic review including 820 patients, only one early death, due to hepatic failure, was reported after SIRT [111].

### 3.4. Contraindication of SIRT

Absolute contraindications for microsphere-based 90Y treatment include HPS >20% or an estimated lung dose >30Gy and reflux into arteries that supply the gastroduodenal region, which may result in extrahepatic deposition of microspheres, and cause non-target radio-induced complications [93,112]. The preSIRT work-up aims to prevent these complications.

Elevated bilirubinemia, pre-existing small liver, intrahepatic biliary duct dilatation, and portal vein obstruction should contraindicate SIRT because of an increased risk of REILD [100]. Other contraindications consist in coagulation abnormalities, renal, heart, or liver decompensated diseases and are similar to those of TAE/TACE.

### 3.5. Expected Therapeutic Response of SIRT for NELM

#### 3.5.1. Overall Survival

No multicentric controlled longitudinal trial has been reported. However, two recent meta-analyses including 19 and 11 retrospective studies, and more than 800 patients with NELM found a median global OS of 28 months (range: 14–70 months) and 32 months (range: 18–57 months) after SIRT [93,111].

#### 3.5.2. Disease Control and Radiological Response

Radiological follow-up is performed using RECIST 1.1 or mRECIST criteria [110,114]. Radiological objective responses rate (including CR and PR) range from 12% to 80% with a weighted mean on meta-analyses of 51% (95% CI: 47–54%) [93,115]. Several studies have reported CR that occurred in 1% to 8% of patients, which is not the case for TAE/TACE [93,110,116,117,118]. After SIRT, treated lesions appear hypovascular or necrotic in 97% of patients [95]. In a recent meta-analysis of 27 retrospectives studies, Frilling et al. found a mean disease control rate (including CR, PR, and SD) of 88% (95% CI: 85–90%) with a median PFS of 41 months [93]. Jia et al. found similar results in a systematic review of 11 studies with a disease control rate of 86% (range: 62.5–100%) [111].

#### 3.5.3. Biological Control

A decrease of 5-HIAA, chromogranin A, and serotonin serum levels were reported in few studies and observed in 35% to 80% of patients after SIRT [95,119,120].

#### 3.5.4. Symptoms and Quality of Life

In a dedicated longitudinal study designed to evaluate the quality of life of patients treated with SIRT for NELM using the SF-36 dedicated questionnaire, Cramer et al. found a significant improvement of mental health and social functioning after 6 months of treatment that could last 24 months [121]. Improvement of symptoms was reported in 20% to 80% of patients with NELM treated with SIRT [106,110,118,119,120,122]. Braat et al. reported a complete regression of symptoms in 35% of those patients [93].

### 3.6. Prognosis Factors

#### 3.6.1. SIRT Parameters

The objective of the dose target remains relatively poorly known for NELM and less studied compared to HCCs [97]. A study found a tumor dose cut-off of 191 Gy as a predictor of response using a per-tumor analysis with an 83% sensitivity, 93% specificity, and 0.89 AUC for the response using RECIST criteria (*p* = 0.038) [97]. On the opposite, a specific tumor dose <73 Gy is associated with a non-response [97].

#### 3.6.2. Post-Therapeutic Imaging Prognosis Factors

A decrease of lesion diameters, low enhancement, and high necrosis portion on post-SIRT MRI compared to pre-SIRT MRI are prognostic factors of survival after SIRT [123,124]. Using molecular imaging, a decrease in liver-to-spleen uptake standardized uptake value (SUV) ratio using 68Ga-DOTATOC PET between pre- and post-SIRT examinations is associated with improved OS and PFS [125]. A complete response using RECIST 1.1 criteria is associated with an improved OS [126].

#### 3.6.3. Clinical and Pathological Prognosis Factors

Low hepatic tumor burden, well-differentiated tumor, female gender, absence of extrahepatic disease, low tumor grade, and Ki67 < 2% are associated with higher OS [94,117,124,126,127]. Pancreatic NETs and unknown primary tumor are associated with a lower OS [110,115]. Pre-SIRT decompensated liver disease and ascites prognosis factors associated with poor OS [94]. An ECOG PS of 0 was associated with an improved OS at multivariate analysis [118].

#### 3.6.4. Biological Prognosis Factors

Preoperative albumin >35 g/L and bilirubin <12 mg/L were positive prognostic factors of OS at univariate analysis [118]. At multivariate analysis, bilirubin serum level <12 mg/L and low neuron-specific enolase (NSE) (cut off: 20.5 μg/L) are predictors of good OS [118,124].

## 4. Comparison of TAE, TACE, and SIRT for the Treatment of NELM

A retrospective study comparing 197 patients treated with TACE to 51 patients treated with SIRT found no differences in grade III/IV morbidity event occurrence (13.7% for SIRT vs. 22.6% for TACE; *p* = 0.17), 30 days mortality (2.0% vs. 3.1%; *p* = 1.0), OS (35.9 months vs. 50.1 months; *p* = 0.3), and PFS (15.9 months vs. 19.9 months; *p* = 0.37) between the two groups [128]. The authors reported a higher rate of outpatients treated with SIRT (92% vs. 1%) that could be interpreted as a higher short-term tolerance rate for SIRT, but this comparison was not performed. Patients treated with TACE had a higher rate of controlled disease using RECIST 1.1 criteria (96% vs. 83%; *p* < 0.01) [128]. Major limitations of this study were the heterogeneity of drugs used for TACE and the absence of consideration of the tumor dose for SIRT [128]. In a retrospective study of 72 patients, SIRT yielded a better 3-year survival rate (88%; 95% CI: 40–90%) than TACE (53%; 95% CI: 10–90%) (OR = 0.1; *p* < 0.036) in patients with a Ki67 > 3% [129].

Withney et al. compared the effectiveness and cost of DEBDOX TACE compared with SIRT in 43 patients and found no differences in OS and higher treatment expense for SIRT (mean cost per session, $25,243; range: $22,227–$40,478) compared to DEBDOX (total mean cost, $13,400; range: $8283–$16,198) (*p <* 0.001). The complications rate tended to be lower for SIRT (9% vs. 18%, *p* = 0.08) [130].

Elf et al. compared TAE to SIRT in a pilot randomized study including 11 patients with midgut NET. The objective response rate was higher in patients treated with TAE (*p* = 0.002), and no significant differences were observed for biological response and complications; however, hospital stay was longer in the TAE group but no OS comparison was performed [108].

## 5. Specificity of NELMs Compared to Other Hepatic Metastases

Conceptually, interventional radiology in the treatment of NELMs differs from that in the treatment of other hepatic metastases. First, NELMs are usually hypervascular tumors with a predominant arterial vascularization. This is why intra-arterial treatments are effective. In this regard, embolization alone can be used for a subset of NELMs (i.e., those from midgut origin) [20] whereas it needs to be associated with intra-arterial chemotherapy for hepatic metastases from colorectal cancer [131]. Second, intra-arterial therapies in NELMs have demonstrated efficacy although there are less effective for metastases of other origins. Finally, NELMs may be associated with biological syndrome due to endocrine secretion. Besides local tumor growth control, one goal of intra-arterial treatment is to control endocrine secretion of NELMs, which is not the case for hepatic metastases from other origins.

## 6. Conclusions

For patients with NELMs who are not candidates for surgery because of high liver involvement or inaccessible locations, or those who are refractory to medical treatment, intra-arterial treatments are safe and effective options to attain disease control, and biological and symptom improvement in selected patients despite a variable tolerance. Experienced interventional radiologists may have a central place during the discussion of patient care. Future large prospective trials are required to define which treatment between TAE, TACE, or SIRT is the most effective one and to improve patient selection, management, and follow-up.
